# Boron Removal by Sorption on Modified Chitosan Hydrogel Beads

**DOI:** 10.3390/ma14195646

**Published:** 2021-09-28

**Authors:** Joanna Kluczka, Gabriela Dudek, Wojciech Pudło, Alicja Kazek-Kęsik, Roman Turczyn

**Affiliations:** 1 Department of Inorganic, Analytical Chemistry and Electrochemistry, Faculty of Chemistry, Silesian University of Technology, B. Krzywoustego 6, 44-100 Gliwice, Poland; alicja.kazek-kesik@polsl.pl; 2 Department of Physical Chemistry and Technology of Polymers, Faculty of Chemistry, Silesian University of Technology, ks. M. Strzody 9, 44-100 Gliwice, Poland; gabriela.maria.dudek@polsl.pl (G.D.); Roman.Turczyn@polsl.pl (R.T.); 3 Department of Chemical Engineering and Process Design, Faculty of Chemistry, Silesian University of Technology, ks. M. Strzody 7, 44-100 Gliwice, Poland; wojciech.pudlo@polsl.pl

**Keywords:** boron, removal, sorption, chitosan hydrogel beads, manganese ions

## Abstract

An excess concentration of boron in irrigation and drinking water can negatively affect the yield of plants and the human nervous system, respectively. To meet the recommended levels, hybrid biosorbent hydrogel beads based on chitosan and manganese (II-IV) were employed for the removal of boron from aqueous media. The results showed that the biosorbent effectively removed boric acid from the aqueous medium at neutral pH over a sorption time of 2 h and the liquid/hydrogel ratio of 20 mL/g, achieving a maximum sorption capacity near 190 mg/g. The modeling of the sorption equilibrium data indicated that the Freundlich isotherm equation gave the best fit out of the isotherm models examined. A pseudo-second-order model was found to best describe the sorption kinetics. The favorable attachment of manganese to the chitosan structure enabled the sorption of boron and was confirmed by FTIR, RS, XRD, SEM and ICP-OES methods. Boron desorption from the spent biosorbent was successfully achieved in three cycles using a NaOH solution. In general, the results of this research indicate that this method is one of the possibilities for improving water quality and may contribute to reducing pollution of the aquatic environment.

## 1. Introduction

Boron is a low-abundance element in the Earth’s crust. As the first element in group 13 of the periodic table, its properties differ significantly from those of the other boron group elements. Boron is a non-metal, occurring in nature in the form of minerals, such as borax and kernite.

In a natural water ecosystem, the concentration of boron is usually small and does not exceed 0.5 mg/L in freshwater. However, increased boron levels can be observed locally due to some natural or anthropogenic factors. Consequently, the amount of boron in waters ranges widely, in groundwater from <0.3 to >100 mg/L (from <0.5 to >1.5 mg/L in EU) and in surface water from <0.001 to even >360 mg/L (from <0.001 to >2 mg/L in EU), which is the case with boron-rich deposit occurrence or wastewater discharges [1]. For most of the world, the concentration of boron in drinking water is judged to be below 0.5 mg/L [2], which is closely related to the amount of boron in fresh waters, which are a principal source of drinking water. In regions with increased volcanic activity or boron mining, such as Turkey, the boron concentration in tap water can be as high as 29 mg/L [3].

Since boron can negatively affect the nervous system in humans and mammals, as well as significantly reduce the yield of plants susceptible to excessive boron, it is recommended to remove it from drinking water, irrigation water, and watercourses down to a concentration limit ranging from 3 mg/L to 0.1 mg/L, depending on the recommendations of the country concerned [4,5].

To meet the above recommendations, scientists have considered many methods, such as sorption, ion exchange, co-precipitation, and membrane techniques, to eliminate boron compounds from water and wastewater. Currently, boron is removed using boron-selective resins (BSRs) by the ion exchange technique, most often in systems integrated with other separation techniques, such as precipitation, reverse osmosis, and electrodialysis [6]. Despite many advantages, the method has a serious drawback, namely high investment and operating costs. Sorption is a superior technique because of its low cost, easy operation, and good yield [6,7]. The interest in biosorbents obtained from plants or animal waste has been growing in recent years [8,9,10,11,12]. In addition to the advantages mentioned above, such biosorbents often require shorter preparation time, are easily available, biodegradable, and do not disturb the balance of the ecosystem. Many researchers have tried to remove boron from water with various biosorbents. Among them, those based on alginate, cellulose, and chitosan deserve special mention [8,10,11].

Chitosan is a biopolymer separated from the skeleton of crustaceans (such as crabs and shrimp) and the cell walls of fungi. Among other biopolymers, chitosan is characterized by its’ easy ability to create various morphological structures, such as films, fibers, hydrogels, membranes, nanoparticles, and microspheres. One of the fabrication methods of chitosan beads is a coagulation process. This method takes advantage of the low solubility of chitosan in alkaline solutions, a polymer that precipitates when in contact with an acidic solution with a basic medium. Depending on the method of producing the droplets, different sizes of spheres can be obtained: macro-, micro-, or even nano-spheres. Other methods of obtaining micro- and nano-spheres include the emulsifying of an acidic aqueous chitosan solution in the oil phase combined with a cross-linking and ion gelation of acidic chitosan solution in a solution of tripolyphosphate polyanions. Chitosan films are obtained by pouring out the solution and gelling it by cross-linking. The choice of fabrication method depends on the specific application. Apart from the chemical structure and biological activity, the variety of structural forms enables the use of chitosan in many areas of the economy: water and wastewater treatment, the food industry, the textile industry, the paper industry, the cosmetics industry, pharmacology, and biomedicine [12]. Various water pollutants, such as metal cations, anions, dyes, and pharmaceuticals, can be captured by the hydroxyl (OH) and amino (NH_2_) functional groups present in the chitosan structure through chelating effects or electrostatic attraction [12,13,14]. Modifying chitosan can make the biopolymer acquire even better properties. One of the modification methods is the cross-linking of chitosan, e.g., with glutaraldehyde, epichlorohydrin, and diethyl ether, which improves mechanical and chemical resistance. Another very popular method is mixing chitosan with other substances, such as polymers, e.g., poly(vinyl alcohol), poly(acrylic acid), collagen, gelatin, cellulose, polylactide, and poly(ethylene oxide) [12].

In recent years, the introduction of many transition metal ions to the chitosan skeleton has been carried out, often giving excellent sorption capacities for anionic pollutants, such as fluoride, nitrate, phosphate, chromate, vanadate, and borate [15,16,17,18,19,20,21]. These hybrid materials can be synthesized by the incorporation and entrapment (or encapsulation) of metal oxide or other metal compound micro- or nano-particles in a chitosan matrix. In water purification, manganese oxides or zeolites modified with them are often used, because in the process of removing impurities, such as arsenic (III) or chromium (III), manganese oxides combine catalytic oxidation and sorption [22]. The efficient removal of arsenic from water using granular sorbent Fe–Mn binary oxide-impregnated chitosan beads was reported by Qi et al. [23]. The separation of Co (II) ions from environmental sample solutions by a MnFe_2_O_4_/bentonite nanocomposite (a magnetic nanomaterial) was described by Rahmani et al. [24]. Muliwa et al. [25] reported manganese removal from water using chitosan/bentonite/MnO composite beads. The removal of nickel from groundwater by iron and manganese oxides was carried out by Matern et al. [26]. Saeed et al. [27] synthesized a manganese oxide-polyvinyl chloride (MnO_2_-PVC) composite for the removal of methylene blue from aqueous solutions. Another way to introduce some transition metal ions to the chitosan skeleton is via direct coagulation of the hybrid material during contact with metal salt dissolved in acidic chitosan solution with a base solution. The authors have reported the use of various cations, such as Ni^2+^, Fe^3+^, Co^2+^, and Zr^4+^, to turn raw chitosan into an effective sorbent for borate [10,19,20,21]. However, until now, boron recovery using manganese, both alone and together with chitosan, has not been comprehensively studied.

Bearing in mind the possibility of using manganese compounds in water and wastewater treatment technology, as well as the favorable results of preliminary studies of boron sorption on freshly precipitated manganese hydroxide, in this study, we designed a sorbent that was a combination of manganese and chitosan. Chitosan acted as a matrix, and its granular form had a task to facilitate the separation of sorbent from the water after the purification process. The purpose of the present study was to characterize the new biosorbent based on a chitosan matrix with trapped manganese (II) ions introduced to the chitosan skeleton by coagulation, to study the equilibrium of boron sorption using this hybrid biosorbent in a batch system, and to determine the possibility of boron desorption and the reusability of this hybrid biosorbent.

## 2. Materials and Methods

### 2.1. Reagents

Chitosan (molecular weight 600,000–800,000) was purchased from Acros Organics (Geel, Belgium); its degree of deacetylation, determined by a ^1^H NMR method, was 97%. Manganese chloride, MnCl_2_·4H_2_O, and other reagents, namely potassium nitrate, sodium hydroxide, and hydrochloric acid, were supplied by Avantor Performance Materials Poland S.A., Gliwice, Poland. A working boron solution of a concentration of 5 g/L was prepared using boric acid provided by Avantor Performance Materials Poland S.A., Gliwice, Poland. A basic standard solution of boron in the form of boric acid (1 g/L) and a manganese standard solution of 1 g/L were supplied by Merck.

### 2.2. Biosorbent Preparation

**Figure 1:**
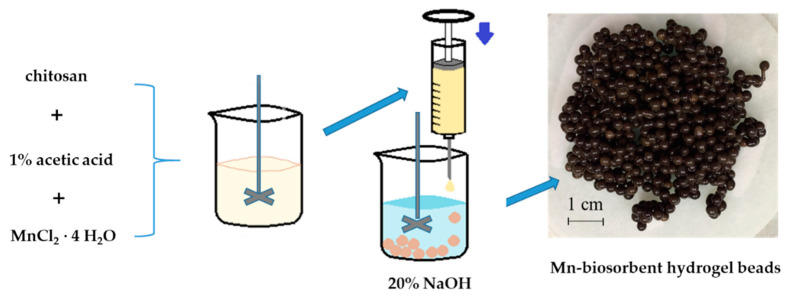
Scheme of synthesis process of the Mn-biosorbent hydrogel beads [28].

The procedure of the Mn-biosorbent hydrogel bead preparation is schematically given in Figure 1 [28]. The procedure is as follows: 3.0 g of pre-washed and dried chitosan was dissolved in 1 wt% acetic acid solution (0.075 L), mixed with 2.7 g of MnCl_2_∙4H_2_O, and stirred for 24 h to obtain a homogenous solution. The mixture was then dripped into a stirred 20 wt.% NaOH solution (0.300 L) through a 0.8 mm syringe needle, forming beads upon contact of the gel drops with the alkaline solution. These formed beads were then kept in the NaOH solution for 24 h, then filtered and washed with demineralized water to remove traces of gelling solution until the rinses reached pH 7.

### 2.3. Analytical Methods and Procedures

The surface morphology of the biosorbent was observed by SEM (scanning electron microscope) using a Phenom ProX SEM (Phenom-World BV, Eindhoven, The Netherlands). The favorable attachment of Mn ions to the chitosan structure was confirmed by FTIR (Fourier-transform infrared) using a Spectrum Two spectrometer (PerkinElmer, Waltham, MA, USA). The RS (Raman spectroscopy) spectra obtained with a spectrometer (inVia confocal Raman microscope, Renishaw plc, United Kingdom) were used to confirm boron sorption on the biosorbent. The phase composition of the biosorbent was determined using a Seifert 3003TT X-ray powder diffractometer with a Cu X-ray tube (Seifert, Ahrensburg, Germany). The specific surface area was determined by low nitrogen adsorption (using a Micromeritics ASAP 2020 adsorption analyzer, Micromeritics Inc., Norcross, GA, USA) using the Brunauer–Emmett–Teller equation (BET equation) [29]. Before the analyses, the chitosan beads were lyophilized using a freeze drier (Christ Alpha 1-2 LDplus, Martin Christ GmbH, Osterode am Harz, Germany) to remove the water from the pore structure. The samples were lyophilized for 70 h at −22 °C with condenser adjustment under a reduced pressure of 0.54 to 0.33 mbar. For the FTIR, RS, XRD, and BET methods, a portion of the dry beads was powdered in an agate mortar. Prior to taking the nitrogen adsorption measurements, each chitosan sample was outgassed for 24 h under vacuum at 333 K.

A point of zero charge on a surface of the biosorbent was determined according to the method of Balistrieri and Murray [30], as described in the previous paper [31]. Briefly, to a series of solutions containing 0.025 L of 0.01 M KNO_3_ with differing pH values from 4 to 9, 0.5 g of the biosorbent was added. After 72 h of equilibration with discontinuous shaking, the pH value of the supernatant liquid was noted using a pH meter (Basic 20+, Crison Instruments, Barcelona, Spain). The point of zero charge (pH_pzc_) of the biosorbent surface was read by graphically plotting ΔpH (the difference between the initial and equilibration pH) against the initial pH. The solution pH at which ΔpH = 0 was the pH_pzc_ of the biosorbent.

The boron and manganese concentrations in the solutions were determined by ICP-OES (inductively coupled plasma optical emission spectroscopy) with a Varian 710-ES spectrometer (Varian, Mulgrave, Victoria, Australia).

### 2.4. Sorption and Desorption Studies

The sorption experiments were carried out with 1 g of the Mn-biosorbent in the form of hydrogel beads and boron solution obtained by appropriately diluting a 5 g/L working solution. The mixture of the boron solution and the biosorbent was agitated at 60 rpm in an orbital shaker. The biosorbent was then filtered from the solution to determine the boron and manganese concentrations in the latter. Each experiment was repeated two or three times to obtain repeatable results.

In the studies on the influence of pH on the sorption efficiency, pH was adjusted in the range of 5 to 10; the solution volume and boron concentration were set at 0.01 L and 20 mg/L, respectively; and the process was carried out for 48 h.

For kinetic studies, 4.0 g of the hydrogel beads was added to a volumetric flask containing 0.100 L of boric acid solution at boron concentrations of 20 or 100 mg/L and pH 7 and subsequently agitated for 0.5–72 h. Aliquots were collected at appropriate time intervals and analyzed in terms of boron and manganese concentrations as described earlier.

During the isotherm experiments, 1 g of biosorbent beads and 0.020 L boron solution with a concentration range of 2–5000 mg/L and a pH adjusted to 7 were placed in contact with each other for 2 h at 20 °C.

The boron sorption capacity (*q* (mg/g)) was calculated using the following equation:

(1) $$q=\frac{\left(C_{0}-C\right)}{m}\times V_{0}$$

where *C_0_* and *C* are the initial and final concentrations of boron in the solution (mg/L), respectively; *V_0_* is the volume of the solution (L); and *m* is the dry mass of the biosorbent (g).

To desorb boron from the spent biosorbent, 1 g of biosorbent beads and 0.020 L of sodium hydroxide solution at a concentration of 0.1, 1.0, or 2.0 mol/L were agitated for 24 or 2 h at room temperature. The boron and manganese concentrations were determined in the resulting aliquots based on three experimental results. The boron desorption efficiency, *D* (%), was calculated using the following equation:

(2) $$D=\frac{q_{D}}{q}\times 100$$

where *q*_D_ is the amount of desorbed boron per dry biosorbent mass (mg/g), calculated using the following equation:

(3) $$q_{D}=\frac{C_{D}}{m}\times V_{\mathrm{NaOH}}$$

where *C_D_* is the final concentration of boron in the solution after desorption (mg/L), *V*_NaOH_ is the volume of sodium hydroxide solution (L), and *m* is the dry mass of the spent biosorbent (g).

Finally, after the desorption of boron, the hydrogel was tested for reuse in two consecutive sorption–desorption cycles using the batch sorption and desorption procedures previously described, with a sorption and desorption time of 2 h and an initial boron concentration of 100 mg/L.

## 3. Results and Discussion

### 3.1. Characterization of the Mn-Biosorbent before and after Boron Sorption

#### 3.1.1. Fourier-Transform Infrared Spectroscopy

The FTIR ATR (attenuated total reflectance) spectra and the detailed description of the unmodified chitosan beads and those filled with manganese particles are shown in the Supplementary Materials (Figure S1). The spectrum of the Mn-biosorbent demonstrated two significant absorption peaks at 600 and 420 cm^−1^ that were characteristic of Mn–O stretching modes in tetrahedral sites, whereas the vibration frequency at 550 cm^−1^ corresponded to the distortion vibration mode of an Mn–O peak in an octahedral environment [32]. In the spectrum of the Mn-biosorbent after boron sorption (Mn-biosorbent-B), a noticeable weakening of the tetrahedral Mn-O peak at 600 cm^−1^ and the octahedral Mn-O band at 550 cm^−1^, which was hardly visibly in the shoulder, was noted, along with the appearance of two signals at 475 and 414 cm^−1^, respectively. This was probably an effect of the interaction with adsorbed boron species.

#### 3.1.2. X-ray Diffraction

**Figure 2:**
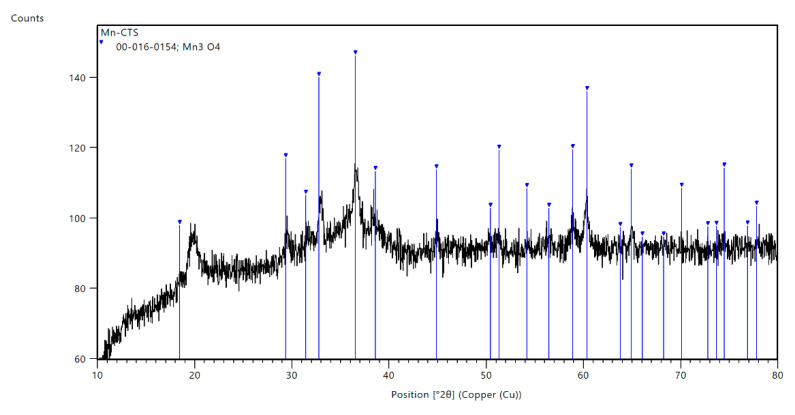
XRD pattern of the Mn-biosorbent.

Figure 2 shows the XRD pattern of the Mn-biosorbent. The sample was in the amorphous crystalline form with a visible broadening of its peaks. The characteristic bump typical for an amorphous phase was recorded in a range from 10° to 25° 2θ and indicated that some amorphous phases existed in the Mn-biosorbent. In this case, the first peak arising from the Mn_3_O_4_ phase was not well defined. The other peaks presented on the XRD pattern corresponded to the Mn_3_O_4_ phase (PDF ref. card: 00-016-0154). The very broad diffraction peaks at 2θ = 20° were typical fingerprints of a semi-crystalline polymer and were related to the crystal-II in the chitosan structure, with a relatively high degree of crystallinity of the polymer matrix [33].

#### 3.1.3. Scanning Electron Microscopy

**Figure 3:**
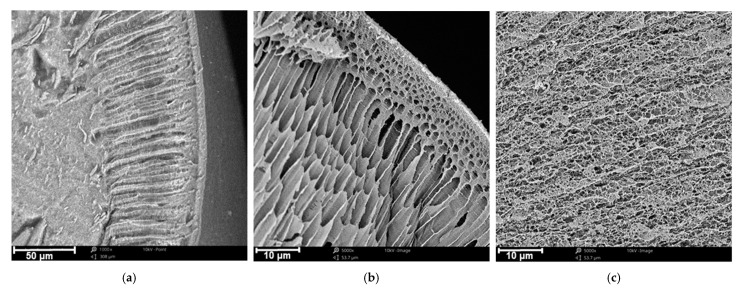
SEM image of a cross-section of the Mn-biosorbent bead: near-surface layer, mag. 1000× (**a**); near-surface layer, mag. 5000× (**b**); and middle area of the bead, mag. 5000× (**c**).

Figure 3 shows the SEM images of the Mn-biosorbent composite bead cross-section. As can be seen, the biosorbent had a porous inner structure with several layers (Figure 3a). The first was a thin, dense skin layer with a size of a few micrometers. Beneath the skin, the layer composed of the channels with a diameter of about 3–4 micrometers and perpendicular to the surface was located (Figure 3b). The interior of the beads was composed of a complex 3D porous network formed by the chitosan matrix (Figure 3c). Such a porous structure greatly facilitated the Mn-biosorbent beads in their sorption of boron throughout the highly developed surface area. As can be seen on the micro-graph (Figure S2b in Supplementary Materials), the structure of the Mn-biosorbent composite bead was not changed significantly after boron sorption; however, the surface of the biosorbent became denser and smoother, which indicates the sorption of boron species in the pores of the Mn-biosorbent bead.

The successful incorporation of the manganese inside the chitosan beads, aside from FTIR spectroscopy, was also confirmed by the EDS elemental analysis conducted during SEM image acquisition. The determined compositions of the unmodified chitosan and Mn-biosorbent beads with incorporated manganese species are presented in Table 1. It should be pointed out that the peak of manganese emerged clearly in the EDS spectrum, confirming that Mn was effectively incorporated into the chitosan hydrogel beads. As could be expected, carbon, nitrogen, and oxygen were the primary elements in the Mn-biosorbent beads, and the content of Mn was approx. 20 wt.%. The sorption of boron ions was not confirmed by EDS due to the limitation of the method associated with its low atomic number.

#### 3.1.4. Specific Surface Area

Nitrogen adsorption isotherms of unmodified chitosan and the Mn-biosorbent are shown in Figure S3 (Supplementary Materials). The adsorption isotherms indicated the presence of a macroporous structure with pores larger than 50 nm (type II isotherm according to the IUPAC classification [34]), which was confirmed by the scanning electron microscope images (Figure 3). In this type of isotherm, a less distinctive Point B (a point that indicates the beginning of the middle, almost linear, section and that usually corresponds to the completion of monolayer coverage) is an indication of a significant amount of overlap of monolayer coverage and the onset of multilayer adsorption [34].

The surface analysis by the BET method [29] shows that the addition of Mn ions to chitosan causes a significant reduction in the specific surface area from 57 to 38 m^2^/g, most probably by creating additional domains of low-porous MnO(OH)_2_ in the chitosan structure [35]. Similar results were given by Demey et al.; the BET-specific surface area of freeze-dried beads prepared using chitosan and iron(III) hydroxide was equal to 37 m^2^/g [19]. However, a surface area of 20% Zr (IV)-doped chitosan estimated by the BET method was found to be 93 m^2^/g [36]. It should be noted, however, that the measurement results of the nitrogen adsorption isotherms depend on the method of sample preparation. In this study, samples were lyophilized by direct freezing in liquid nitrogen at −22 °C before measurement. In [37], the samples were cooled in two stages before freezing, and the results of the specific surface areas were completely different. This means that S_BET_ can only be considered relatively; i.e., the changes before and after modification of the chitosan hydrogel measured according to the same procedure can be compared.

#### 3.1.5. Raman Spectroscopy

**Figure 4:**
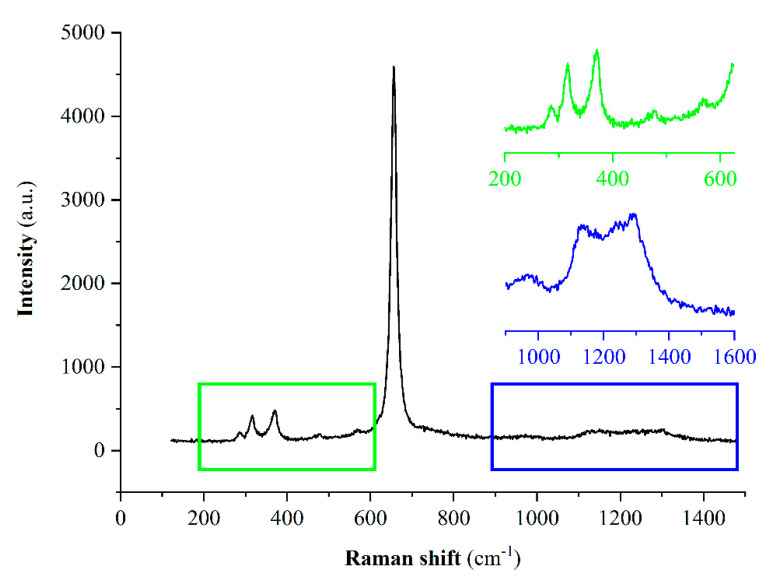
Raman spectra for the Mn-biosorbent after boron sorption.

The presence of adsorbed boron species was confirmed by RS. In Figure 4, the Raman spectrum of the biosorbent after boron sorption is presented. The most intense sharp Raman signal at about 656 cm^−1^ and the three smaller peaks at about 286, 316, and 370 cm^−1^ were characteristic for the spectrum of tetragonal hausmannite Mn_3_O_4_ [38]. However, looking at the enlarged region between 200–650 cm^−1^ and 900–1600 cm^−1^, peaks originating from the adsorbed boron species could be observed. The broad absorptions in the regions 1150–1350 cm^−1^ were regarded as evidence for borates. The small bands at 477 and 567 cm^−1^ possibly suggested the presence of some polyborate species, such as [B_5_O_6_(OH)_4_]^−^ [39].

#### 3.1.6. The Proposed Mn-Biosorbent Structure and Boron Sorption Mechanism

The FTIR and XRD spectra showed that the obtained Mn-biosorbent contained Mn–O bonds, with manganese being present in oxidation states II–IV in the tetrahedral and octahedral configuration. Confirmed spatial structures, octahedral and tetrahedral, could indicate the presence of such manganese coordination compounds as [Mn(H_2_O)_6_]^2+^, [MnCl_6_]^4−^, and [MnCl_4_]^2−^, respectively. However, these compounds are characterized by a low stability in solution and quickly darken due to oxidation [40], which was observed during hydrogel preparation and storage. Hence, tetragonal hausmannite Mn_3_O_4_ (Mn^II^Mn^III^_2_O_4_ spinel) and other hydrated manganese oxides of variable composition, such as Mn(OH)_2_, MnO(OH), and MnO(OH)_2_, which showed a high affinity for boric acid, can be expected in the hydrogel. Based on the SEM images and nitrogen adsorption isotherms, we found the Mn-biosorbent hydrogel beads had a macroporous structure with several layers. Such a porous structure greatly facilitated the Mn-biosorbent beads in their high sorption of boron. As a result of the sorption process, some polyborate species, such as [B_5_O_6_(OH)_4_]^−^, were stated in chitosan, which was confirmed by the RS analysis.

### 3.2. Effect of pH

**Figure 5:**
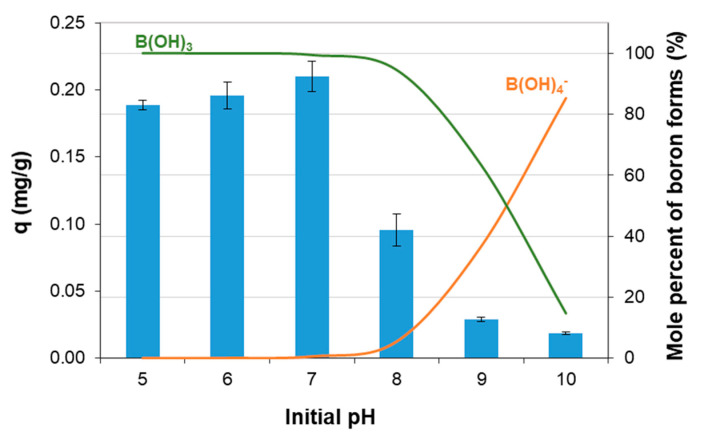
Boron sorption capacity (blue bars) and boron speciation (percentage of boric acid—green line, percentage of borate anion—orange line) as a function of initial pH value; mass of the Mn-biosorbent hydrogel beads: 1 g; initial concentration of boron: 20 mg/L; volume of solution: 10 mL; contact time: 48 h; temperature: 20 ± 1 °C.

A very significant parameter, which influences the fractionation of boron chemical forms in water, is the pH of the aqueous solution. Boric acid, B(OH)_3_, and tetrahydroxyborate, B(OH)_4_^−^, are the main chemical forms of boron in natural water [1]. The concentration of both molecules and anions is determined by the equilibrium state between B(OH)_3_ and B(OH)_4_^−^, which is described by the first dissociation constant pK_a_ of boric acid, which is equal to 9.28 in pure water at a temperature of 20 °C [41]. As can be seen in Figure 5, at a pH below 7, mainly boric acid molecules were present in the solution (green color line). Both forms occurred in the pH range of 7–11, with anions predominating above pH 9.28 (orange color line).

**Figure 6:**
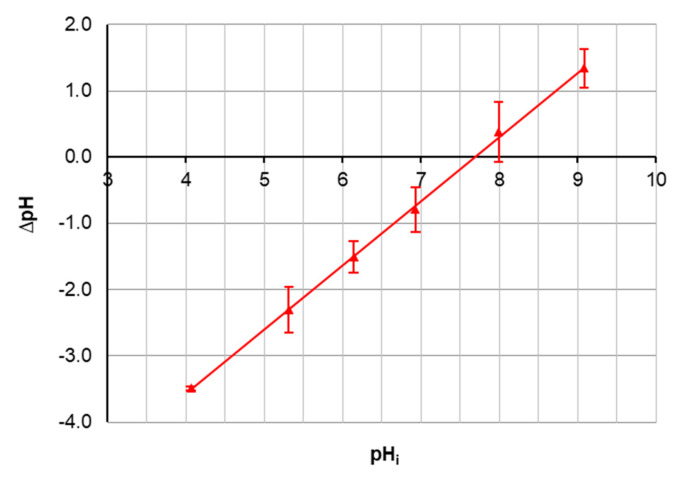
pH_i_ versus ΔpH for the Mn-biosorbent hydrogel beads.

The possible presence of boron in the solution in the forms of a molecule or a complex anion means that an important parameter in the process of boron sorption is the surface charge of the sorbent, the so-called point of zero charge on a surface (pH_pzc_). This point of zero charge means that the surface of the sorbent has a positive charge below pH_pzc_ and a negative charge above pH_pzc_. The determined pH_pzc_ value of the Mn-biosorbent was 7.7 ± 0.3 (Figure 6). In the case of the adsorptive removal of anions from the aqueous solution, it is desirable for the sorbent surface to be positively charged. Accordingly, at pH < 7.7, the surface of the Mn-biosorbent was positively charged, and B(OH)_4_^-^ anions, which are negatively charged, could be electrostatically attracted to the hydrogel surface.

The influence of the solution’s pH on boron sorption by the Mn-biosorbent is illustrated in Figure 5 (blue bars). When the pH was in the range of 5 to 7, the sorption capacity remained rather constant; a slight increase up to 0.210 ± 0.014 mg/g was observed at a pH of 7. At pH values higher than 7, boron sorption on the Mn-biosorbent was drastically decreased, which might be explained by the repulsive force between the negatively charged B(OH)_4_^−^ species that dominated in the alkaline solution and the negative charge of the sorption sites on the surface of Mn-biosorbent that occurred at pH > pH_pzc_ (7.7). As a result, the boron sorption was depressed with a further increase in the pH value to above 8.

### 3.3. Kinetic Study

**Figure 7:**
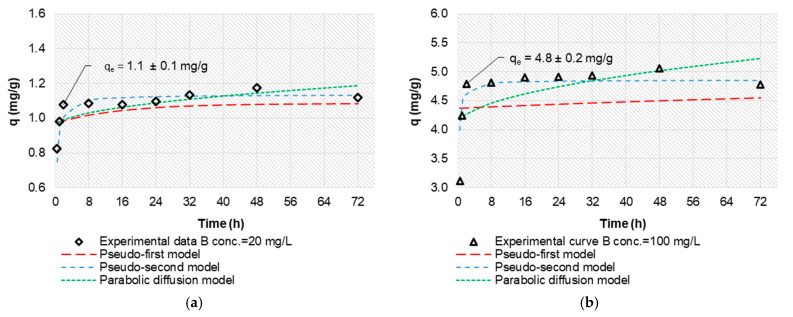
Effect of contact time on boron sorption efficiency using Mn-biosorbent hydrogel beads and boron sorption fit with kinetic and diffusion model; initial boron concentration: 20 mg/L (**a**) and 100 mg/L (**b**).

The influence of contact time, *t*, on boron sorption capacity, *q*, is shown in Figure 7. An equilibrium state was achieved after 2 h for the initial concentrations of boron 20 mg/L and 100 mg/L. To determine the kinetics of sorption, three kinetic models, namely the pseudo-first-order, the pseudo-second-order, and the parabolic diffusion models, were tested [42,43,44]. The pseudo-first-order kinetic model is given by Equation (4):

(4) $$q_{t}=q_{e}\times \left(1-\mathrm{Exp}\left(-k_{1}\times t\right)\right)$$

where *q_t_* and *q_e_* are the amounts of boron sorbed (mg/g) at any time *t* and at the time of equilibrium, and *k_1_* is the pseudo-first-order rate constant for the sorption process (1/h). The pseudo-second-order kinetic equation is expressed by Equation (5):

(5) $$\frac{t}{q_{t}}=\frac{1}{k_{2}\times {\left(q_{e}\right)}^{2}}+\frac{t}{q_{e}}$$

where *k_2_* is the pseudo-second-order rate constant (g/(mg × h)). Moreover, an initial sorption rate, *r* (mg/(g × h)), at *t* = *0*, can be calculated using the constant *k*_2_ according to Equation (6):

(6) $$r=k_{2}\times {\left(q_{e}\right)}^{2}$$

The overall kinetics of the sorption from solutions may be governed by diffusional processes as well as by the kinetics of the surface chemical reaction. In diffusion studies, the rate is often expressed in terms of the square root of time (Equation (7)):

(7) $$q_{t}=k_{p}\times t^{1/2}$$

where *k_p_* is the intraparticle diffusion rate constant (mg/(g/(h)^1/2^)).

The parameters of the three kinetic models and their correlation coefficients, *R*^2^, for two initial boron concentrations are listed in Table 2. The calculated values of *R*^2^ for the pseudo-first-order kinetic and the intraparticle diffusion equations were very low, indicating that these models are not applicable to the discussed process. For the pseudo-second-order kinetic model, the coefficients *R*_2_^2^ were 0.999, which meant a good correlation between this model and the experimental data. The calculated values of *q*_2_ agreed with the experimental sorption capacity, and the initial sorption rate, r, increased when the initial boron concentration increased from 20 to 100 mg/L.

The results indicated that the pseudo-second-order model gave an excellent description of boron sorption on the solid surfaces of the Mn-biosorbent hydrogel beads for initial concentrations of boron ≤ 100 mg/L. This meant that the sorption rate of boron species in hydrogel beads could be controlled by the chemisorption. We suggest the non-homogeneous process was caused by the inseparability of the transport phenomena and the chemical reactions. A similar case was also noted by other researchers; e.g., the sorption of copper ions in chitosan granules also proceeded according to a mixed mechanism combining the chemical reaction with diffusion inside the porous structure of chitosan [45].

### 3.4. Sorption Isotherms

**Figure 8:**
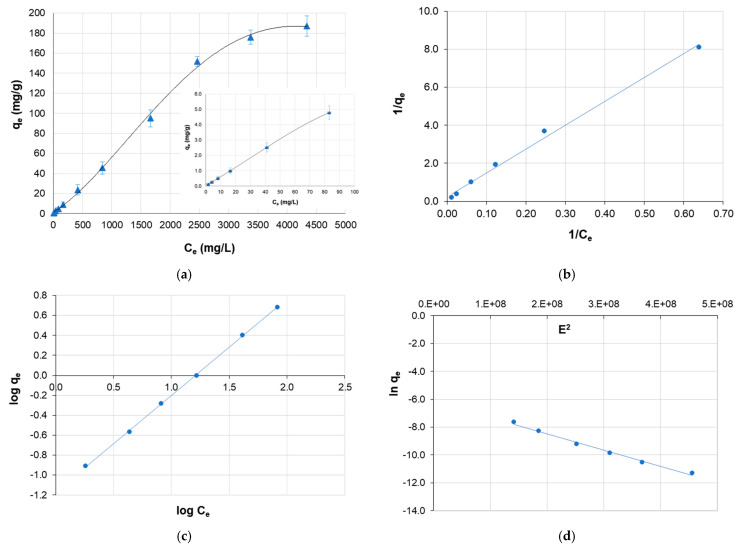
Sorption isotherms of boron on the Mn-biosorbent hydrogel beads at 20 °C: sorption capacity vs. equilibrium boron concentration; initial boron concentration range: 10–5000 mg/L and 2–100 mg/L (the inserted figure) (**a**); boron sorption data fitted with Langmuir model (**b**); boron sorption data fitted with Freundlich model (**c**); boron sorption data fitted with Dubinin–Radushkevich model (**d**); pH: 7.0; hydrogel dose: 1 g/20 mL.

The sorption capacity of the Mn-biosorbent for boron was evaluated by isothermal experiments, and the results are shown in Figure 8. The Mn-biosorbent was characterized by a high sorption capacity for boron; the experimental sorption capacity achieved a value of 187 ± 10 mg/g at 20 °C and a pH value of 7, with a contact time of 2 h and an initial boron concentration of C_0_ = 5 g/L. As can be seen in Figure 8a, the sorption isotherm was regular, positive, and slightly convex to the consistency axis. The initial slope was steep (see the inserted figure in Figure 8a showing the isotherm in the concentration range 2–100 mg/L), indicating that the affinity of the hydrogel beads to boron is strong. Such an isotherm was classified as the L-class and the 1-subgroup according to the classification of Giles et al. [46].

To describe the experimental data, three two-parameter models of isotherms were applied: the Langmuir, Freundlich, and Dubinin–Radushkevich equations [47,48,49]. The Langmuir model (Equation (8)) supposes a homogeneous surface with regard to the energy of sorption, which is equal for each surface site; no interaction between the sorbed species; and an equal availability of each sorption site to all the sorbed species.

(8) $$q_{e}=q_{m}\times \frac{B\times C_{e}}{1+B\times C_{e}}$$

where q_e_ is the amount of boron adsorbed at equilibrium (mg/g), C_e_ is the concentration of boron in the solution at equilibrium (mg/L), and *q_m_* and *B* are the Langmuir parameters: *q_m_* is the sorption capacity (mg/g), which is equal to the maximum amount of boron that can be sorbed by the biosorbent as a monolayer, and *B* is an equilibrium constant that corresponds to the sorption energy (L/mg). The Freundlich model (Equation (9)) is applicable to the sorption process that occurs on heterogeneous surfaces on which there are several kinds of sorption sites, each having a different energy:

(9) $$q_{e}=K\times C_{e}{}^{1/n}$$

where the parameters *K* ((mg/g)(L/mg)^1/n^) and *n* relate to the sorption capacity and the sorption intensity of the biosorbent, respectively. The Dubinin–Radushkevich (Equation (10)) equation is a local isotherm in which the sorption follows a micropore filling mechanism:

(10) $$q_{e}=x_{m}\times e^{-\left(k\varepsilon^{2}\right)}$$

where *ε* is the Polanyi potential, which is equal to *RT*ln (1 + 1/*C_e_*); *x_m_* is the sorption capacity (mg/g); *k* is a constant corresponding to the sorption energy (mol^2^/kJ^2^); *T* is the temperature (K); and *R* is the gas constant (kJ/(mol × K)). From the Dubinin–Radushkevich model, the sorption energy can be calculated according to the following equation:

(11) $$E=-{\left(2k\right)}^{-\left(0.5\right)}$$

The calculated parameters and correlation coefficients are presented in Table 3. The experimental data in the range of the initial boron concentration 2–100 mg/L fitted the Freundlich equation better than the Langmuir and Dubinin–Radushkevich models because the correlation coefficient (R^2^) was 0.9996. Although the Langmuir model gave the high value of correlation coefficient (R^2^ = 0.9950), the calculated value of the parameter *q*_m_ = 4.1 mg/g (sorption capacity of a monolayer) differed from the experimental capacity *q*_exp_ = 4.8 mg/g, which excluded the full interpretation of the process by the Langmuir model. In contrast, the Freundlich model gave the highest value of the correlation coefficient R^2^ and a parameter *n* greater than 1, which indicated favorable sorption and showed this model to be adequate for describing the discussed process [50]. Furthermore, the Freundlich model usually corresponds to the sorption process on heterogeneous surfaces, which confirms the conclusions of the kinetic studies. As can be seen in Table 3, the Dubinin–Radushkevich model was less suitable because of the slightly lower correlation coefficient. The calculated value of *X_m_* (the sorption capacity of the micropores) equaled 21.8 mg/g and was not in agreement with the experimental sorption capacity (4.8 mg/g). The calculated value of the sorption energy (*E*), less than 20 kJ/mol, could mean that the sorption of boron on the Mn-biosorbent proceeded by means of weak Van der Waals forces [50].

### 3.5. Desorption of Boron

**Figure 9:**
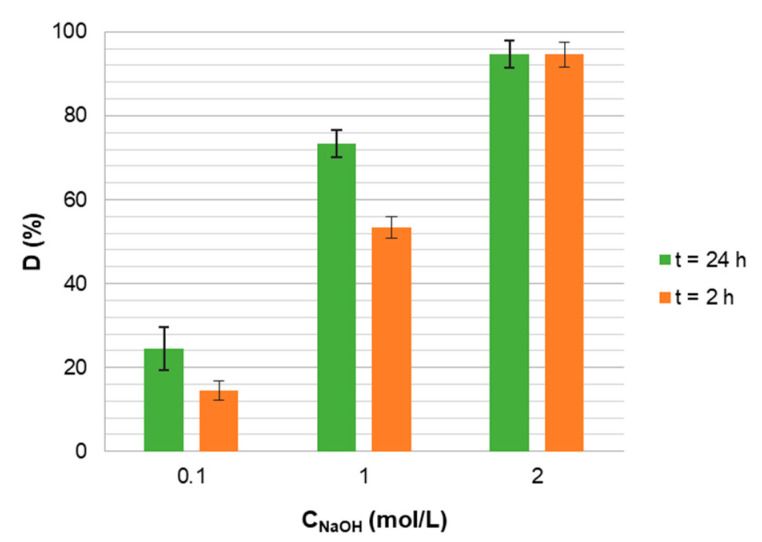
The influence of the concentration of NaOH solution and time on boron desorption efficiency from the spent Mn-biosorbent hydrogel beads.

Based on the boron sorption and capacity results presented in Figure 5, which show that the sorption capacity of boron on the Mn-biosorbent proceeded very weakly under alkaline conditions, we decided to conduct the desorption experiment in a sodium hydroxide solution. The influence of the NaOH concentration and contact time on boron desorption efficiency, *D* (%), is shown in Figure 9. Satisfactory boron desorption, 95 ± 3%, was observed using 2 mol/L NaOH solution after both 24 and 2 h.

### 3.6. Reuse and Stability of the Mn-Biosorbent

Sorption–desorption cycles were repeated three times, with a sorption and desorption time of 2 h to determine the reusability of the biosorbent. As can be seen in Table 4, the sorption capacity slightly decreased in the second and third cycles. The quantitative desorption yield in the first cycle was 94% when over 100% in the second and third cycles. Because the stability of the biosorbent, especially the bond strength between the filler and chitosan, is a significant factor for qualifying the proposed biosorbent for use in purifying water or wastewater [51], we checked the leaching of manganese ions from the Mn-biosorbent during the sorption and desorption process. The results shown in Table 4 indicated that trace amounts of manganese eluted within sorption and desorption to the solution, but the Mn concentration was far less than the legal limit of 0.5 mg/L (the limit for manganese concentration is 0.5 mg/L, both in natural water and in wastewater discarded into the environment [51,52]). Furthermore, we noticed that a compact form of hydrogel beads was retained after three sorption–desorption cycles (see Figure S4 in the Supplementary Materials).

**Figure 10:**
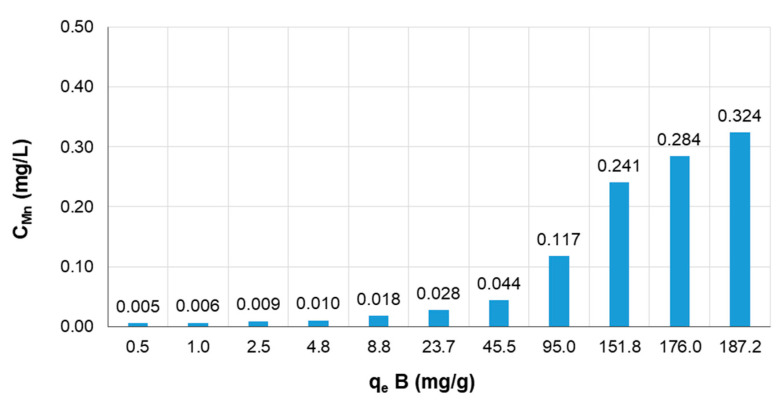
The influence of the sorption capacity of boron on the elution of manganese ions from the Mn-biosorbent hydrogel beads. Initial boron concentration C_0_ = 100–5000 mg/L; biosorbent dose 1 g/20 mL; temperature 20 °C; contact time 2 h.

We also checked the sorbent behavior when it was immersed in high concentration solutions of boron, up to 5 g/L. The elution of manganese ions from the biosorbent into the solution after 2 h of sorption is shown in Figure 10. The concentration of manganese in the solution after sorption was dependent on the sorption capacity, and it increased with increasing q_e_. However, the C_Mn_ in the solution was relatively low, even for the highest sorption capacity 187 mg/g and was 0.324 mg/L. This result means that the leaching of manganese ions was below the legal limit of 0.5 mg/L and that there was no secondary manganese contamination of the treated water.

### 3.7. Comparison of the Results

As mentioned in the introduction, there are many papers on boron removal from water and wastewater in the available literature, among them are those dealing with removal by sorption. This is due to, on the one hand, quite restrictive boron concentration limits in force in some countries and, on the other hand, the need to treat water due to its global scarcity. The result obtained in our research, 187 mg of boron per 1 g of dry Mn-biosorbent (equivalent to a sorption capacity of 13 mg B per 1 g of wet hydrogel), is a very high result, which makes the proposed manganese–chitosan hydrogel competitive to BSRs (a maximal capacity between 5.9 and 7.2 mg-B/g [53]). Table 5 compares the sorption capacities of the Mn-biosorbent and other sorbents reported over the last five years.

## 4. Conclusions

We prepared an economical, harmless, and recyclable Mn-biosorbent, employing an encapsulation method. The resulting biosorbent in the form of hydrogel beads was characterized by its structure and behavior in solution and used for the effective elimination of boron from aqueous media.

Based on FTIR, XRD, ICP-OES, and EDS analyses, it was found that manganese (II) ions in the new biosorbent were incorporated into the chitosan lattice and that they were oxidized to MnO(OH) and MnO(OH)_2_. The surface of the biosorbent was macroporous with several layers, however, with a much smaller specific surface area relative to the surface area of the raw chitosan hydrogel (which we determined by the BET method and observed using an SEM microscope). The reduction in the BET surface indicated that the pores were filled with manganese compounds and that the chemical properties, rather than the physical properties, of the surface were of dominant importance for boron sorption.

Thus, the favorable attachment of manganese to the chitosan structure enabled the sorption of boron. The presence of sorbed boron, probably as polyborates, was confirmed by ICP-OES and RS analysis. At pH 7 and within about 2 h, we obtained a maximum sorption capacity of 187 mg/g. The modeling of the experimental data indicated the best fit occurred for the Freundlich isotherm equation and a pseudo-second-order kinetic model, which indicated that the sorption process was heterogeneous. We suggest a mixed mechanism combining the chemical reaction with diffusion inside the granule structure of Mn-biosorbent best describes this process.

Furthermore, a satisfactory boron desorption, of around 95%, was observed after elution with 2 mol/L NaOH solution. The sorption capacity slightly decreased in the second and third cycles; however, the quantitative desorption yields in the following cycles were satisfactory. No significant trace of Mn ions was determined in the eluate when the sorption and desorption proceeded within 2 h.

The high sorption capacity and successful regeneration of the Mn-biosorbent, its stability under the sorption and desorption process, and beads that are suitable for separation from aqueous media should enable its application in water treatment. Furthermore, the use of sodium hydroxide as a desorbing agent is economically viable. After desorption, the resulting solution can be concentrated and then crystallized as borax, which is an innovative approach to boron recovery from liquid media.

## Figures and Tables

**Table 1:** The element content in the unmodified chitosan and Mn-biosorbent bead determined by the EDS analysis.

Element	Elemental Composition in wt.%	
	Unmodified Chitosan	Mn-Biosorbent
carbon	40.2	19.7
oxygen	38.2	42.2
nitrogen	21.6	17.8
manganese	-	20.2

**Table 2:** Kinetic parameters for boron sorption on the Mn-biosorbent hydrogel beads at 20 ± 1 °C.

Initial Boron Concentration	Experimental Capacity	Parameters of the Kinetic Models			
		Pseudo-first order			
C_0_ (mg/L)	q_expt_ (mg/g)	q_1_ (mg/g)	K_1_ (g/(mg × h))	R_1_^2^	
20	1.08	0.167	−0.006	0.133	
100	4.79	0.439	0.040	0.386	
		Pseudo-second order			
C_0_ (mg/L)	q_expt_ (mg/g)	q_2_ (mg/g)	k_2_ (g/(mg × h))	R_2_^2^	r (mg/(g × h))
20	1.08	1.14	3.41	0.999	4.40
100	4.79	4.86	1.90	0.999	44.8
		Intraparticle diffusion model			
C_0_ (mg/L)	q_expt_ (mg/g)	q_3_ (mg/g)	K_p_ (mg/(g × (h)^1/2^))	R_3_^2^	
20	1.08	0.950	0.028	0.552	
100	4.79	4.07	0.136	0.377	

**Table 3:** Langmuir, Freundlich, and Dubinin–Radushkevich isotherm parameters for boron sorption on the Mn-biosorbent hydrogel beads at 20 ± 1 °C.

Langmuir Model			Freundlich Model			Dubinin–Radushkevich Model		
*q_m_* (mg/g)	*B* (L/mg)	R^2^	*K*_F_ ((mg/g)(L/mg)^1/n^)	*n*	R^2^	*x*_m_ (mg/g)	*E* (kJ/mol)	R^2^
4.1	0.0195	0.9950	0.068	1.035	0.9996	21.8	−7.1	0.9914

**Table 4:** Sorption and desorption efficiency of the Mn-biosorbent hydrogel beads.

Cycle No	q (mg/g)	D (%)	Manganese Concentration in Solution (mg/L)	
			After Sorption	After Desorption
1	4.44 ± 0.20	94.5 ± 2.2	0.010 ± 0.002	0.026 ± 0.002
2	4.17 ± 0.18	103.4 ± 3.8	0.027 ± 0.008	0.020 ± 0.002
3	4.07 ± 0.07	102.2 ± 3.1	0.023 ± 0.008	0.020 ± 0.001

**Table 5:** Comparison of various boron sorbents reported in 2016–2021.

Sorbent/Parameter	Temp. (°C)	pH	C_0_ (mg/L)	Sorbent Dosage (g/L)	t (h)	q (mg/g)	Ref.
BSR Amberlite IRA-743	30	9.5	40	10	-	7.5	[6]
Alginate–alumina	25	9.5	500	7	72	19.6	[8]
Alumina	25	9.5	500	7	72	4.5	[8]
Chitosan–NanoTiO_2_	25	4	20	50	5	4.3	[10]
Chitosan–NanoCr_2_O_3_	25	4	20	50	5	3.5	[10]
Chitosan–NanoFe_3_O_4_	25	4	20	50	5	4.4	[10]
Chitosan–Fe(OH)3	25	4	20	50	5	7.8	[10]
Chitosan–NanoTiO_2_	25	4	20	50	5	4.3	[10]
Chitosan–Co(OH)_2_	25	8.5	20	50	60	2.5	[31]
Activated carbon F400	25	7	60	10	2	0.85	[54]
F400-mannitol	25	8.5	60	10	4	1.5	[54]
Waste tire rubber	-	2	17.5	-	-	16.7	[55]
Glycidol-magnesium ferrite	25	7	100	1	0.5	69.2	[56]
Polyethylenimine-epichlorohydrin resin	25	9	5000	5	24	55	[57]
Polyurethane-algae	-	7	6.44 OPW *	20	72	0.27	[58]
Cooper oxide nanoparticles	25	7	10	1	24	3.5	[59]
Chitosan–Ce(OH)_4_	20	7	500	100	48	13.5	[60]
Chitosan–La(OH)_3_	20	5	100	100	24	11.1	[61]
Poly (VBC-DVB)-TRIS	-	8	500	-	-	8.99	[62]
Nano-magnetite (Fe_3_O_4_)	-	8	50	0.5	3	8.44	[63]
Mn-biosorbent	20	7	5000	50	2	187	Present study
Mn-biosorbent	20	7	100	50	2	4.8	Present study

* Oil-produced water.

## Data Availability

Data is contained within the article or supplementary material.

## Supplementary Materials

The following are available online at https://www.mdpi.com/article/10.3390/ma14195646/s1, Figure S1: FTIR spectra of the unmodified chitosan, the Mn-biosorbent and the Mn-biosorbent after boron sorption (Mn-biosorbent-B) [28], Figure S2: SEM image of a cross-section of the Mn-biosorbent bead (a) and SEM image of the Mn-biosorbent after boron sorption (Mn-biosorbent-B) (b), Figure S3: Nitrogen adsorption isotherms for unmodified chitosan and Mn-biosorbent, Figure S4: Photo of the Mn-biosorbent hydrogel beads just after the third boron sorption cycle completed.
