# Supplementary material for: Boron Removal by Sorption on Modified Chitosan Hydrogel Beads
Source: Materials (Basel). 2021 Sep 28;14(19):5646. doi: 10.3390/ma14195646 (PMC8510040; doi:10.3390/ma14195646)
Supplement: Supplementary file 1 [file materials-14-05646-s001.zip › materials-1350953-supplementary.pdf]

materials

*Supplementary Materials*

# Boron Removal by Sorption on Modified Chitosan Hydrogel Beads

**Joanna Kluczka, Gabriela Dudek, Wojciech Pudło, Alicja Kazek-Kęsik and Roman Turczyn**

### Fourier-Transform Infrared Spectroscopy Results and Discussion

Figure S1 shows the FTIR spectra of the unmodified chitosan beads and those filled with manganese particles. The spectrum of pure chitosan beads showed several characteristic bands. The bands at 1652 and 1596 cm$^{-1}$ corresponded to the -C=O stretching of the amide I from non-deacetylated amine groups and the N-H bending of the amide II, respectively. The -$CH_2$ bending and -$CH_3$ symmetrical deformations gave bands at around 1420 and 1381 cm$^{-1}$, respectively. The band at 1151 cm$^{-1}$ was attributed to asymmetric stretching of the C–O–C bridge of the ether group. The bands at 1077 and 1028 cm$^{-1}$ corresponded to the C-OH stretching of the secondary hydroxyl group, and the C-OH and C–$NH_2$ stretching of the primary hydroxyl and amine groups, respectively. Additionally, a carbon ring breathing mode peak at 944 cm$^{-1}$ and an amine group out-of-plane (N–H wagging) peak at 894 cm$^{-1}$ were noted. The Mn-biosorbent demonstrated two significant absorption peaks at 600 and 420 cm$^{-1}$ that were characteristic of Mn–O stretching modes in tetrahedral sites, whereas the vibration frequency at 550 cm$^{-1}$ corresponded to the distortion vibration mode of Mn–O peak in an octahedral environment [29]. In the spectrum of Mn-biosorbent-B, a noticeable weakening of the tetrahedral Mn-O peak at 600 cm$^{-1}$ and the octahedral Mn-O band at 550 cm$^{-1}$, which was hardly visibly in the shoulder, was noted, along with the appearance of two signals at 475 and 414 cm$^{-1}$, respectively. This was probably an effect of the interaction with adsorbed boron species. The bands originated from the vibrations of B-O bonds, which were expected in the 1200-1350 cm$^{-1}$ and 900 cm$^{-1}$ regions, were not observed on the spectrum due to the overlapping with signals originated from a polymer matrix.

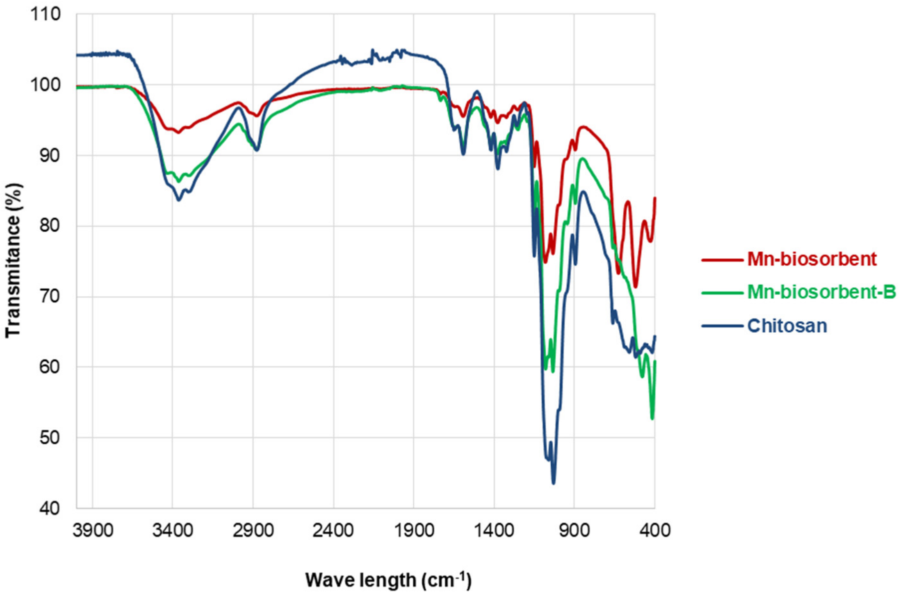


**Figure S1.** FTIR spectra of the unmodified chitosan, the Mn-biosorbent and the Mn-biosorbent after boron sorption (Mn-biosorbent-B) [28].

## Scanning Electron Microscopy results

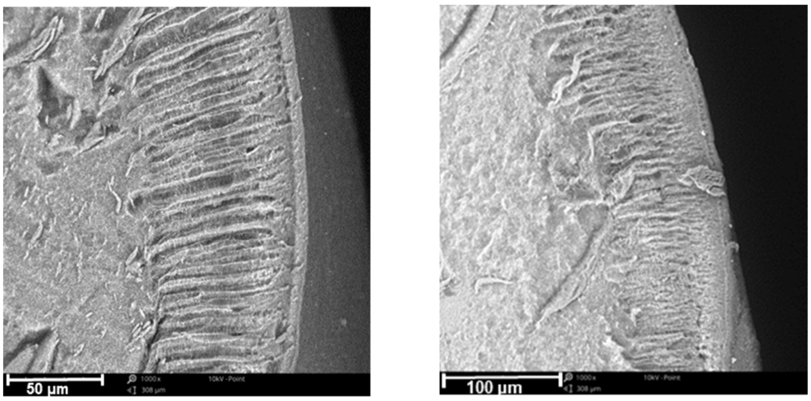


(**a**) (**b**)

**Figure S2.** SEM image of a cross-section of the Mn-biosorbent bead (**a**) and SEM image of the Mn-biosorbent after boron sorption (Mn-biosorbent-B) (**b**).

## Specific Surface Area Results

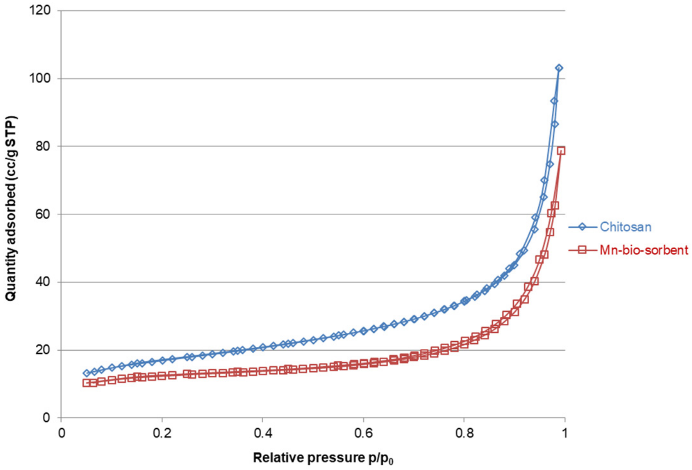


**Figure S3.** Nitrogen adsorption isotherms for unmodified chitosan and Mn-biosorbent.

## The Stability of the Mn-Biosorbent Hydrogel Beads

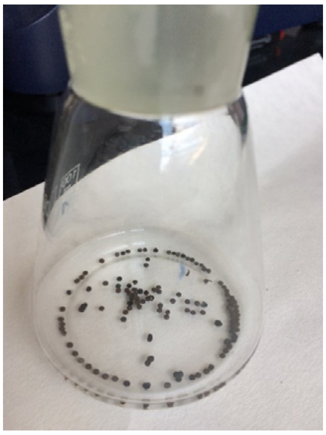

**Figure S4.** Photo of the Mn-biosorbent hydrogel beads just after the third boron sorption cycle completed.
